# Performance of national HIV testing algorithms in 14 Population-based HIV Impact Assessment surveys: accuracy of HIV diagnosis using a two-test strategy, with or without a tie-breaker test, in different prevalence settings, 2015-2022

**DOI:** 10.1128/jcm.01173-25

**Published:** 2025-10-30

**Authors:** Hetal K. Patel, Yen T. Duong, Sehin Birhanu, Melissa Metz, Jared Garfinkel, Kathryn Lupoli, Daniel Yavo, Herbert Longwe, Faith Ussery, Kristin Brown, Stephen McCraken, Clement B. Ndongmo, Jessica Justman, Andrew C. Voetsch, Bharat S. Parekh, for the PHIA Laboratory Working Group

**Affiliations:** 1 Division of Global HIV & TB, CGH, CDC 119199 , Atlanta, Georgia, USA; 2 ICAP at Columbia University 5798, New York, USA; Mayo Clinic Minnesota, Rochester, Minnesota, USA

**Keywords:** HIV, testing algorithm, survey, laboratory testing, quality, PHIA

## Abstract

Population-based HIV Impact Assessment surveys were conducted in 14 countries to measure HIV prevalence, incidence, and viral load suppression in children aged 0–14 years and adults aged ≥15 years. We examined the performance of rapid HIV testing algorithms in multiple countries. Survey participants received an HIV diagnosis based on two serial tests in Cameroon, Côte d’Ivoire, Eswatini, Haiti, Kenya, Lesotho, Malawi, Mozambique, Tanzania, and Zambia. Whereas in Ethiopia, Kenya, Namibia, Uganda, and Zimbabwe, diagnosis was based on a tie-breaker algorithm. Testers received intensive training on specimen collection, testing, and results reporting. HIV-positive and indeterminate results were confirmed by Geenius HIV-1/2 test (Bio-Rad). Pooled test 1 and 2 positive concordance, test 2 and 3 negative concordance, and the positive predictive value (PPV) of the algorithm were calculated. A total of 312,069 participants aged ≥18 months (except for Eswatini, Mozambique, and Uganda, where only adults aged 15+ years were included) were tested by either the two-test (*n* = 243,311) or tie-breaker (*n* = 98,757) algorithm for diagnosis. The combined test 1 and 2 positive concordance and PPV were 93.8% and 99.4%, respectively, for countries with a two-test algorithm. Similarly, test 1 and 2 positive concordance, test 2 and 3 negative concordance, and PPV were 87.5%, 84.4%, and 98.8%, respectively, for the tie-breaker algorithm. The PPV ranged from 94.2% to 99.9%, with higher PPV in high burden countries. The HIV testing algorithms performed with high accuracy, with PPV reaching 99% or higher. Adoption of continuous quality improvement is essential to ensure accuracy of HIV diagnosis in service delivery settings.

**IMPORTANCE:**

HIV diagnostic testing in most African countries follows national algorithms that typically use two tests, with or without a tie-breaker. We assessed the accuracy of these algorithms using data from population-based surveys in 14 sub-Saharan African countries, where all HIV-positive results were further confirmed with the Geenius HIV-1/2 supplemental assay. Our findings show that inter-test concordance and positive predictive values (PPVs) varied by HIV prevalence, with higher PPVs observed in higher-prevalence settings. Overall, the PPV of HIV diagnosis was close to 99%, indicating that two-test algorithms can provide highly accurate results when testing is performed with strict adherence to quality standards and tester competency. These results underscore the importance of quality assurance (QA) and suggest that countries with lower HIV prevalence may benefit from adopting a three-test algorithm. However, such changes should be accompanied by careful attention to logistics, procurement, training, record keeping, and other QA measures.

## INTRODUCTION

Accurate HIV diagnosis is a cornerstone of HIV treatment programs and can be achieved through various testing methodologies and strategies. Point-of-care rapid HIV tests (RHTs) can be conducted clinics, hospitals, community centers, and home self-testing, allowing for rapid decision-making and the opportunity to provide timely prevention and treatment services (1–3). These point-of-care assays are integrated into a structured algorithm, which typically includes a series of steps and methods to establish a national rapid HIV testing algorithm. Traditionally, the national HIV testing algorithms consisted of either two serial tests (Test 1 [T1] and Test 2 [T2]) or two serial tests (T1 and T2) followed by a tie-breaker test (T3) in case of discordant results, and they are well established in most sub-Saharan African countries (4).

RHT was first introduced in sub-Saharan Africa in the late 1990s. Despite early concerns about stigma, discrimination, diagnostic challenges, complex laboratory systems, and limited treatment availability, HIV programs have expanded significantly over the past two decades, thanks to access to HIV testing with widespread use of RHT. Today, more than 83 million people undergo testing annually using the national HIV testing algorithms (5, 6). Overall, ease of use, immediate availability of test results, accessibility, convenience, and linkage to treatment have played a crucial role in promoting HIV awareness, ensuring access to lifesaving treatment, ultimately reducing mortality, and preventing transmission of HIV. With the expansion of RHT use, the number of sites and staff providing services has also increased. Although RHTs are user-friendly, countrywide implementation requires proper training, continuous review of competency/certification requirements, proper documentation, and quality control testing. Many quality assurance (QA) tools, such as rapid test continuous quality improvement training packages, proficiency testing using dried tube specimens, and results recording in standardized logbooks, are available for President’s Emergency Plan for AIDS Relief (PEPFAR) programs (7). However, there is still a lack of systematic large-scale site-level data on individual test performance and comprehensive reviews of the national testing algorithm performance.

More than 25 RHTs are included on the World Health Organization (WHO) prequalification list of diagnostic assays, and 6–8 are included on the U.S. Food and Drug Administration (FDA) list (8). Both WHO and FDA have minimum performance requirements of ≥99% sensitivity and ≥98% specificity (9). In addition, other performance criteria (inter-reader variability, invalid rates), operational characteristics (ease of use, test packaging, and ease of result interpretation), and regulatory auditing of manufacturing facilities are reviewed during the approval process (9). The pre-qualification list is utilized by the ministries of health to select the most appropriate rapid tests for use in testing algorithms within their HIV programs.

Since 2014, the Population-based HIV Impact Assessment (PHIA) surveys, nationally representative household surveys that measure HIV prevalence and incidence, have been conducted in multiple PEPFAR-supported countries. Additionally, these surveys have been standardized and implemented in multiple countries to measure the UNAIDS 95-95-95 targets: the first 95, which represents the percentage of people living with HIV who know their HIV status; second 95, which represents the percentage of those who are aware of their HIV status who are receiving antiretroviral treatment; and the third 95, which represents the percentage of people on treatment who have a suppressed viral load (VL), as established by the Joint United Nations Programme on HIV/AIDS (10). To accurately measure the entire set of targets, the first 95, or the percentage of people living with HIV who know their HIV status, reflecting the accuracy of HIV diagnosis, is critical.

During PHIA surveys, eligible participants were tested and received the results in their homes using the national rapid HIV testing algorithm. Additionally, for QA processes, laboratory-based confirmation using a supplemental assay was done to examine the performance of the national rapid HIV testing algorithm in 14 PHIA surveys, namely, Cameroon, Côte d’Ivoire, Eswatini, Ethiopia, Haiti, Kenya, Lesotho, Malawi, Mozambique, Namibia, Tanzania, Uganda, Zambia, and Zimbabwe. These countries used their respective testing algorithms in HIV testing services (HTSs) to diagnose HIV infection; however, supplemental assays were not routinely used to further confirm algorithm accuracy. Use of supplemental testing in PHIA surveys provided an opportunity to evaluate national HIV testing algorithm performance in these countries using testing data from these surveys. Findings from this analysis may have implications in HTSs, where the same strategy is commonly used.

## MATERIALS AND METHODS

### PHIA study design

The PHIA project study objectives, study design, sample size, eligibility criteria, and other survey details have been described elsewhere (11). Briefly, the PHIA countries and their respective survey years described in this report are as follows: Cameroon (2017–2018), Côte d’Ivoire (2017–2018), Eswatini (2021), Haiti (2019–2020), Kenya (2018–2019), Lesotho (2016–2017), Malawi (2015–2016), Mozambique (2021–2022), Tanzania (2016–2017), Zambia (2016), Ethiopia (2017–2018), Namibia (2017), Uganda (2020–2021), and Zimbabwe (2015–2016). For each national survey, consenting adults (15+ years, upper age band varied by country) and children (18 months to 14 years, except for Eswatini, Mozambique, and Uganda surveys) underwent HIV counseling, and they provided blood for rapid HIV testing and provided answers to an interview administered by field staff in the household (HH). Participants provided blood samples for HIV testing and other biomarkers as described in each study protocol, including HIV confirmation, HIV recency testing, CD4+ count, VL levels, and detection of antiretroviral and drug resistance for those blood samples testing HIV-positive.

### Specimen collection and processing

In all countries, a phlebotomy-trained nurse/laboratorian collected whole blood specimens from study participants during the HH visit. Depending on age, approximately 1 mL was collected from children under 2 years using a heel prick and an ethylenediaminetetraacetic acid (EDTA) microtainer (Becton Dickinson, USA), 6 mL from children aged 2–14 years using a single 6-mL EDTA vacutainer, and 14 mL total from adults 15+ years in a 10-mL and 4-mL EDTA vacutainer. For participants where venous blood draw was not possible (collapsed veins or no consent for venous blood draw), approximately 1 mL of blood was collected using a finger prick and an EDTA microtainer. Once the HH testing was completed, specimens were collected and transported in coolers with freezer packs to satellite or central labs. They were processed into plasma and/or dried blood spots (DBSs), tested as needed, and stored within 24 h of collection time to ensure specimen integrity for all testing (12).

### HIV testing algorithms and testing

For each PHIA, the country’s national rapid HIV testing algorithm, either two test or two test with a tie-breaker, was used to establish the objective HIV status of study participants (aged 18 months to 15+ years). All RHT was performed in the participant’s HH, and results from individual rapid tests dictated whether additional RHT was required. All individual biomarker test kit information (lot number and expiry date) and test results (as reactive, non-reactive, or invalid) were recorded in the pre-programed survey tablets. All RHTs used in the surveys were on WHO’s prequalification list. The Determine HIV-1/2 (Abbott, Illinois, USA) was T1 in all countries, except for Tanzania, where SD-Bioline HIV-1/2 (Abbott, Illinois) was used, and Ethiopia, where Wantai HIV-1/2 (Beijing, China) was used. HIV testing was performed according to the manufacturers’ instructions using the whole blood sample. The following three outcomes were recorded for the two-test algorithms: participants with a non-reactive T1 result were reported as negative. Those with reactive results for both T1 and T2, conducted serially, were reported as positive. If T1 was reactive and T2 was non-reactive, the algorithm was repeated. If results remained discordant upon repeat testing, they were reported as indeterminate. For the tie-breaker algorithms, the first two outcomes remained unchanged. However, if T1 was reactive but T2 was non-reactive, T3 was performed. HIV status was determined from the results of T3 (reactive or non-reactive), with the result reported as HIV-positive or HIV negative, respectively, based on the results of the tie-breaker test. While in Uganda, the results of T3 distinguished HIV indeterminate from HIV negative; for comparability, the results of T3 reactive were considered HIV-positive in this analysis. Final HIV diagnosis was immediately returned to the participant at the HH and recorded in survey tablets, referral forms, and sample tracking forms. Additionally, participants received pre- and post-test counseling, and those with HIV-indeterminate results were referred for further testing at a healthcare facility of their choice. Participants who tested HIV-positive were informed that HIV VL testing would be conducted, and the results would be provided later to the health facility of their choice.

### Quality management and confirmatory testing

To ensure test kit performance, bi-weekly external quality control panels (HIV-positive and negative) were tested by all field and laboratory staff. QA testing was performed at the satellite laboratory (usually district hospital laboratories) or central laboratories (usually national reference laboratories). The QA testing strategy involved retesting the first 25–50 samples (25 for Eswatini, Uganda, and Mozambique and 50 for all other countries) tested by each field tester using the country testing algorithm as performed in the HH, to ensure ongoing testing proficiency. In addition, for most countries, 5% of HIV-negative specimens, as well as all HIV-indeterminate specimens, were also retested. HIV confirmatory testing of all HIV-positives (and in some cases of all HIV indeterminates) was conducted using Geenius HIV-1/2 Confirmatory Assay (Bio-Rad, USA) according to the manufacturer’s instructions (13–15). The assay is a rapid immunochromatographic test that has the capacity to distinguish between HIV-1 and HIV-2 infection. The Geenius HIV-1/2 assay was not part of any national RHT algorithm of the participating countries. For some cases where repeat Geenius testing was required and whole blood or plasma was not available, an in-house validated DBS elution protocol was used, which was previously used in another study (16).

### Data review and final classification

 Final HIV classification for each survey was based on results from national testing algorithms conducted at the HH and confirmatory testing using Geenius HIV-1/2 at the laboratory. The Geenius HIV-1/2 assay is a more specific test and can potentially distinguish between HIV-1 and HIV-2 antibodies (13). In case of discrepant results between HH testing, QA retesting, and Geenius, specimens were re-tested at the central laboratory. All discrepant results were reviewed case-by-case and adjudicated for additional testing. In very rare cases, when results could not be resolved through retesting, then a HH revisit for re-collection and testing was conducted (data from re-visits are excluded from the analysis presented here). Data were separately analyzed for adults (15+ years) and younger age groups (18 months to 14 years) to allow analysis of testing algorithms in populations with a range of HIV prevalence from low to high. Agreement between the two RHTs was calculated from concordant results, while the positive predictive value (PPV) of the testing algorithm was calculated using final Geenius results as the reference. All results presented are unweighted except country-specific HIV prevalence (17).

## RESULTS

Among 10 countries that used a two-test algorithm (without a tie-breaker test), there were 194,199 eligible adult participants (ages 15+ years); 17,774 (9.2%) of their blood specimens had an HIV-positive test result and 1,175 (0.6%) had an HIV-indeterminate test result (Table 1). Among the four countries that used a two-test algorithm with a tie-breaker, there were 82,062 adult participants; 8,003 (10.8%) had blood specimens with an HIV-positive test result (two out of three reactive tests) (Table 1). Uganda’s country-specific algorithm classified T1 and T3 positive as indeterminate; however, for the purpose of this analysis, they are grouped with the other three countries using the tie-breaker test, where T1 and T3 reactive were classified as HIV-positive.

**TABLE 1:** Summary of total participants tested by the national testing algorithms, either two-test or tie-breaker algorithm, in 14 countries PHIA survey from 2015 to 2022^*b*^

			Ages 15+ years					Ages 18 months to 14 years			
	Country	Survey year	Ages (years)	Total participants	HIV positive	HIV negative	HIV indeterminate	Total participants	HIV positive	HIV negative	HIV indeterminate
Two-test algorithm	Cameroon	2017–2018	15–64	25,985	977 (3.8%)	24,665 (94.9%)	343 (1.3%)	6,564	14 (0.2%)	6,472 (98.6%)	52 (0.8%)
	Cote d'Ivoire	2017–2018	15–64	17,804	466 (2.6%)	17,188 (96.5%)	150 (0.8%)	4,302	21 (0.5%)	4,244 (98.7%)	34 (0.8%)
	Eswatini	2021	15+	11,187	2,878 (25.7%)	8,293 (74.1%)	16 (0.1%)	–^*c*^	–	–	–
	Haiti	2019–2020	15–64	17,718	336 (1.9%)	17,297 (97.6%)	85 (0.5%)	3,161	29 (0.9%)	3,115 (98.5%)	17 (0.5%)
	Kenya	2018–2019	15–64	27,596	1501 (5.4%)	26,046 (94.4%)	49 (0.2%)	9,870	81 (0.8%)	9,764 (98.9%)	14 (0.1%)
	Lesotho	2016–2017	15–59	11,678	3,176 (27.2%)	8,481 (72.6%)	21 (0.2%)	3,651	77 (2.1%)	3,567 (97.7%)	4 (0.1%)
	Malawi	2015–2016	15–64	17,101	2,120 (12.4%)	14,870 (87.0%)	111 (0.6%)	5,673	91 (1.6%)	5,552 (97.9%)	23 (0.4%)
	Mozambique	2021–2022	15+	14,462	2,044 (14.1%)	12,286 (85.0%)	132 (0.9%)	^*c*^–	–	–	–
	Tanzania	2016–2017	15+	31,564	1,839 (5.8%)	29,518 (93.5%)	207 (0.7%)	8,553	40 (0.5%)	8,501 (99.4%)	11 (0.1%)
	Zambia	2016	15–59	19,104	2,437 (12.8%)	16,606 (86.9%)	61 (0.3%)	7,338	80 (1.1%)	7,245 (98.7%)	5 (0.1%)
	**Sub-total**			**194,199**	**17,774** (**9.2%**)	**175,250** (**90.2%**)	**1,175** (**0.6%**)	**49,112**	**433** (**0.9%**)	**48,460** (**98.7%**)	**160** (**0.3%**)
Tie-breaker algorithm	Ethiopia	2017–2018	15–64	19,136	620 (3.2%)	18,516 (96.8%)	–	4,274	17 (0.4%)	4,256 (99.6%)	0 (0.0%)
	Namibia	2017	15–64	16,939	2,439 (14.4%)	14,500 (85.6%)	–	5,968	71 (1.2%)	5,893 (98.7%)	1 (0.0%)
	Uganda	2020–2021	15+	25,413	1565 (6.2%)^*a*^	23,848 (93.8%)	–	–	–	–	–
	Zimbabwe	2015–2016	15–64	20,574	3,379 (16.4%)	17,195 (83.6%)	–	6,453	111 (1.7%)	6,339 (98.2%)	1 (0.0%)
	**Sub-total** ^*d*^			**82,062**	**8,003** (**9.8%**)	**74,059** (**90.2%**)	**–**	**16,695**	**199** (**1.2%**)	**16,488** (**98.8%**)	**2** (**0.0%**)
**All countries**	**Total**			**276,261**	**25,777** (**9.3%**)	**249,309** (**90.2%**)	**1,175** (**0.6%**)	**65,807**	**632** (**1.0%**)	**64,948** (**98.7%**)	**162** (**0.2%**)

^*a*^ Includes 1390 as test 1 and test 2 reactive and 175 as test 1 and test 3 reactive. Uganda survey had a tie-breaker algorithm, however their national testing algorithm classified T1 and T3 reactive as indeterminate. To be consistent with other 3 countries, T1/T3 are considered as HIV-positives for this analysis.

^*b*^ Data here are presented by age groups, 15 years and older and 18 months to 14 years, and by total participants tested as HIV-positive, negative, and indeterminate.

^*c*^ “–” indicates no value.

^*d*^ Boldface indicates subtotal or total of specific column.

HIV-positivity rate among children (18 months to 14 years) was 0.9% for both two-test (433 out of 49,112 tested) and tie-breaker (199 out of 16,695 tested) algorithms.

**Fig 1:**
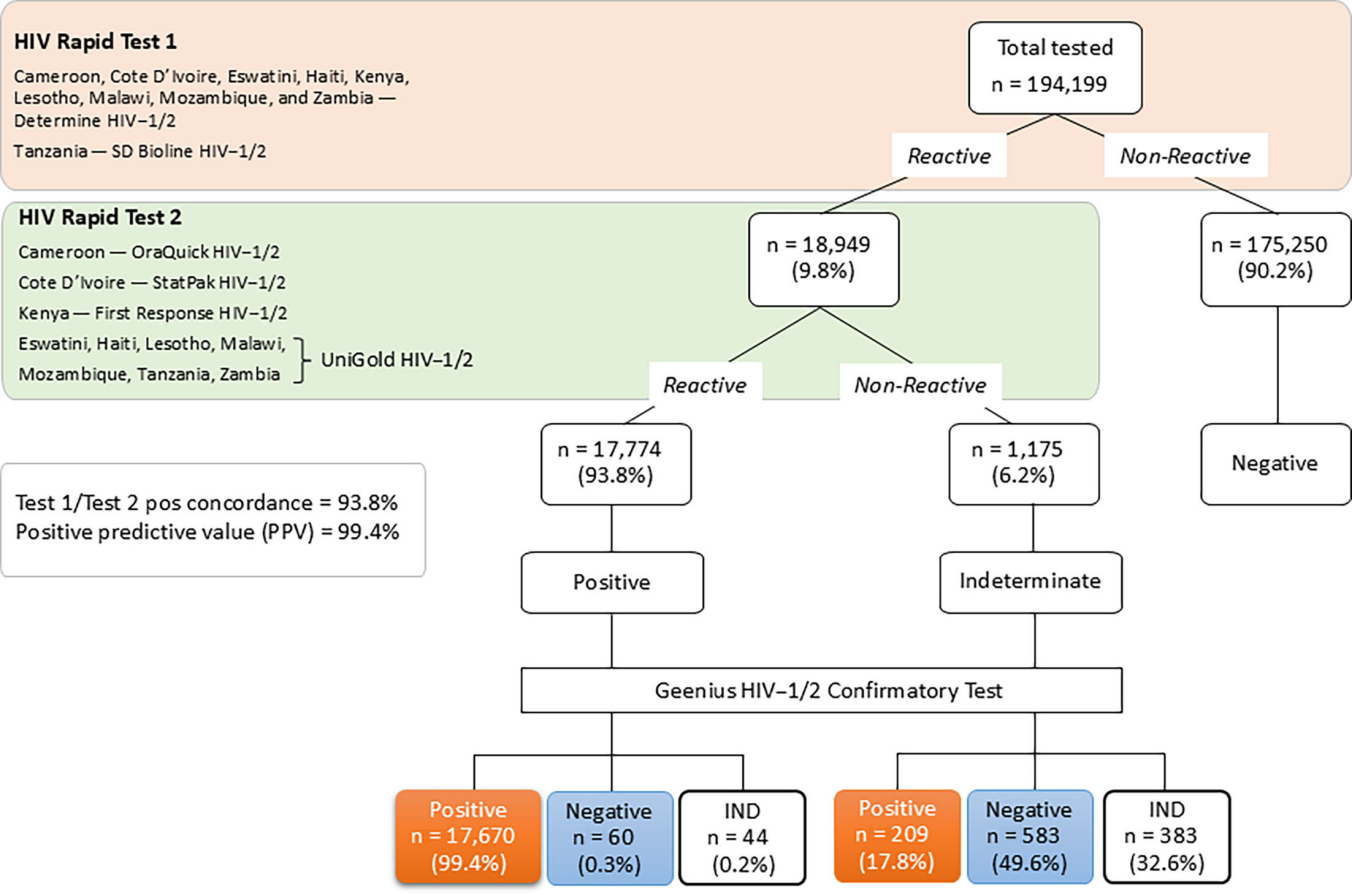
Combined HIV results, aged 15 years and older, from 10 PHIA surveys conducted from 2015 to 2022. Country-specific national HIV testing algorithm, where up to two HIV rapid tests, in serial, are conducted for a final HIV diagnosis. All initial indeterminate results (T1 reactive and T2 non-reactive) were repeated, and participants with repeatedly indeterminate results were asked to seek the health care facility for re-testing.

A total of 194,199 eligible household participants, age 15 years or older, were tested in 10 countries, as part of the PHIA surveys using each country’s respective national two-test algorithm (Fig. 1), nine using Determine HIV-1/2; the tenth, Tanzania, used SD Bioline HIV-1/2. Of the 18,949 (9.8%) participants had blood specimens reactive by T1, 17,774 (93.8%) specimens were confirmed as HIV-positive and 1,175 (6.2%) as HIV indeterminate by T2 per national testing algorithm. Geenius HIV-1/2 rapid test further confirmed 17,670 (99.4%) specimens as HIV positive. Accordingly, the T1 and T2 HIV-positive concordance was 93.8% and the PPV was 99.4% for the two-test algorithm.

**Fig 2:**
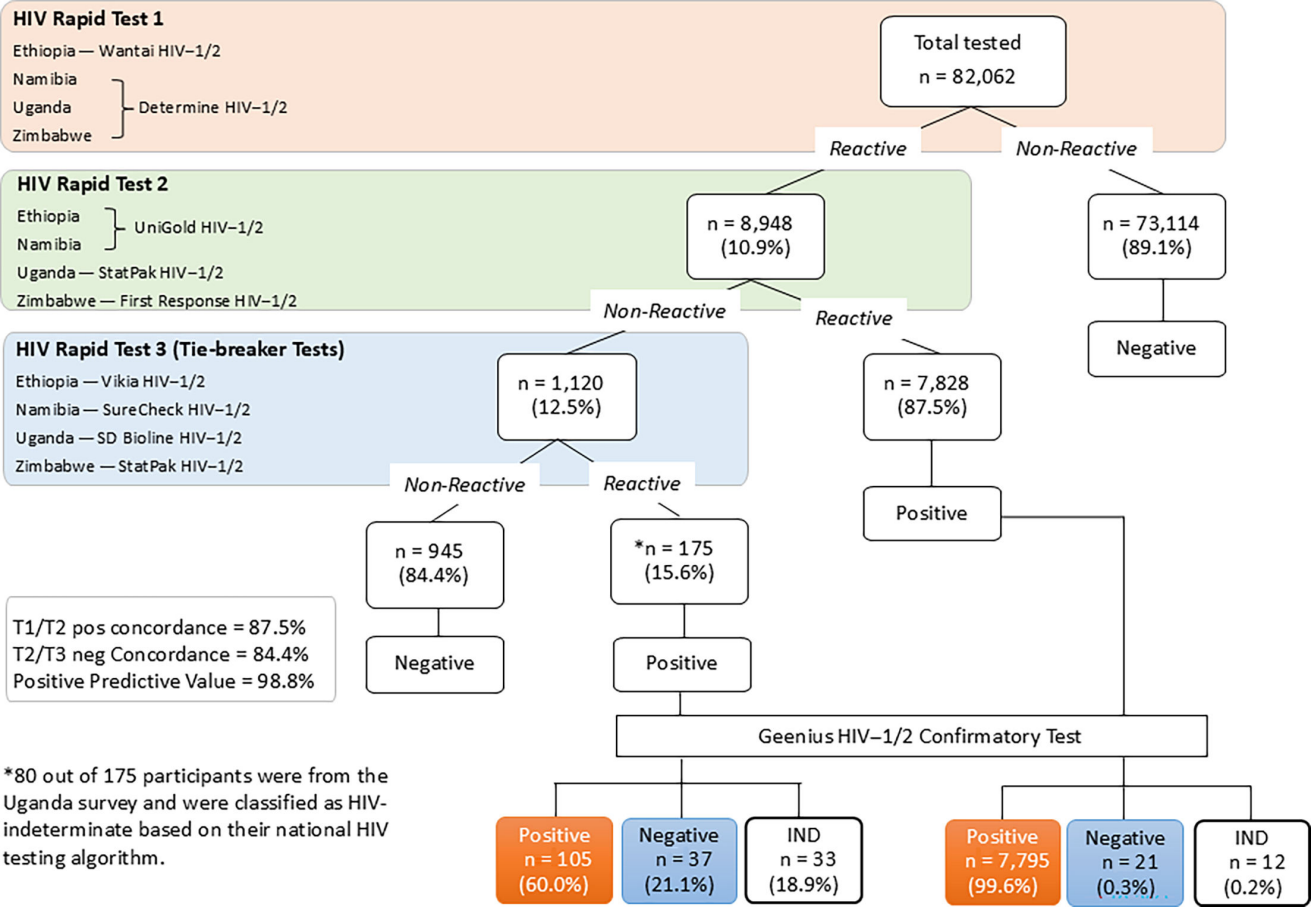
Combined results, from participants aged 15 years and older, from four PHIA surveys, Ethiopia, Namibia, Uganda, and Zimbabwe, using the national two-test algorithm and the tie-breaker HIV testing algorithm. Country-specific national HIV testing algorithm, where three HIV rapid tests, in serial, are conducted for a final HIV diagnosis.

A total of 82,062 participants (ages 15 years and older) had blood specimens tested by a tie-breaker algorithm in four countries; of these, 8,948 (10.9%) were reactive by T1, and 7,828 (87.5%) were reactive and 1,120 (12.5%) were non-reactive by T2 (Fig. 2). Of the 1,120 (12.5%) T2 non-reactive specimens, 175 (15.6%) were reactive by the T3 tie-breaker test, while 945 (84.4%) were non-reactive. The T1 and T2 HIV-positive concordance was 87.5%, and the T2 and T3 negative concordance was 84.4% of the tie-breaker test algorithm. PPVs were separately calculated for T1 and T2 positive and T1 and T3 positive diagnoses, as confirmed by Geenius. A total of 7,795 out of 7,828 T1 and T2 reactive cases were confirmed as positive by Geenius, yielding a PPV of 99.6%, while only 105 of 175 T1 and T3 positive cases were positive on Geenius, yielding a PPV of 60%. This lower PPV was heavily influenced by the Uganda survey where only 12 (15%) of 80 T1 and T3 positive cases were confirmed by Geenius; we note that per the Uganda national algorithm, these participant specimens were considered HIV indeterminate. For the other three countries that used the tie-breaker algorithm, 93 (98%) of 95 T1 and T3 reactive specimens were confirmed as positive by Geenius. Overall, the PPV of the tie-breaker test algorithm was 98.8%.

**Fig 3:**
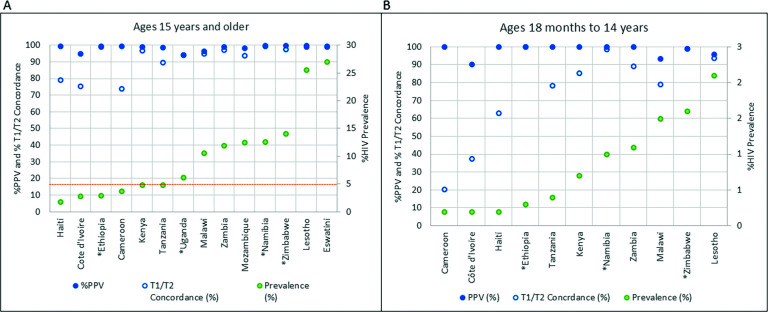
PPV of the testing algorithm and concordance between HIV rapid test 1 (T1) and test 2 by country (x-axis), arranged by increasing HIV prevalence (weighted prevalence). Panel (**A**): ages 15 years and older; panel (**B**): ages 18 months to 14 years. Note: The broken line in Fig. 3A marks a prevalence cut-off of 5%. The scale of the secondary y-axis (%HIV prevalence) in Fig. 3A and B is different for better visualization. Panel (**B**) excludes Eswatini, Uganda, and Mozambique since their surveys did not include <15-year-olds. Additionally, the weighted %HIV prevalence (shown in green dots) is for 0 to 14 years instead of ages 18 months to 14 years in panel (**B**).

The T1 and T2 concordance and PPV of the national testing algorithms by country are presented by HIV prevalence in Fig. 3. The HIV prevalence ranged from 1.8% in Haiti to 27.0% in Eswatini for ages 15+ (Fig. 3A). T1 and T2 concordance ranged from 74.0% to 99.5% and increased with HIV prevalence, whereas the PPV of the national testing algorithm ranged from 95.0% to 99.9%. For children aged 18 months to 14 years (Fig. 3B), T1 and T2 concordance ranged from 20.6% to 100% and RHT algorithm PPV ranged from 90.5% to 100%, increasing with HIV prevalence. Only 632 participants in the 18-month to 14-year age group had specimens with HIV-positive test results; overall T1 and T2 concordance and PPV were highly variable for this relatively small group, especially when disaggregated by country (Fig. 3B). Figure 3B excludes data from Eswatini, Uganda, and Mozambique since their survey did not include <15-year-olds.

## DISCUSSION

Our comprehensive review of the national HIV testing algorithms across multiple PHIA surveys provided several important insights on algorithm performance. The T1 and T2 concordance and PPV of the national testing algorithm increased with HIV prevalence. Across surveys, the average discordance of 11% for both the two test and the tie breaker algorithm strategies, and discordance was even higher in low burden countries. This pattern aligns with previously published findings from Nigeria [18]. The PPV of national testing algorithms for adults aged 15 years and older was >98% in most countries when compared with the Geenius confirmatory assay, except for Côte d’Ivoire (95.1%) and Malawi (96.5%). The PPV for two test and tie-breaker algorithms was similar within this age group. Including a tie-breaker 3rd test to resolve indeterminate cases identified additional HIV-positive cases. The PPV among indeterminate cases was only 60% as confirmed by Geenius, although 85% (68 of 80 were negative by Geenius) of unconfirmed cases were from the Uganda survey. The HIV prevalence among those between 18 months to 14 years was low and as expected the testing algorithm PPVs were lower in this population. We found that a positive result in a low prevalence population may be less reliable and may require additional testing. This finding was consistent with the 2019 WHO HIV testing service (HTS) guidance document which recommends 3-test algorithms to increase the PPV for the test-and-treat era [4].

The 2019 WHO HIV testing strategy encourages both high and low burden countries to use three consecutive reactive test results for HIV-positive diagnosis. While high burden countries, where HIV prevalence is ≥5%, can continue using two consecutive reactive test results to define an HIV-positive outcome, as countries approach or exceed the first 95 goal, residual HIV prevalence of undiagnosed persons will be less than 5%. In such cases, WHO recommends deploying the 2019 three-test algorithm (4). Consequently, as countries move to implement new HIV testing strategies, careful planning and thorough evaluation are essential for the success of their programs. Potential challenges that may arise include, but are not limited to, staff training, logistics and procurement, updates to national guidelines, records and documentation, and the provision of QA, including immediate corrective actions. Additionally, most HTS sites currently use capillary whole blood specimens obtained via fingerprick for RHT. Under the three-test algorithm, discordant results (i.e., T1 reactive and T2 non-reactive) require repeat testing, which often necessitates an additional fingerprick for specimen collection. Moreover, WHO also recommends retesting HIV-positive individuals before ART is initiated, requiring an additional round of testing. Given that the implementation of a new WHO three-test algorithm requires significant resources, we highly recommend programs to assess the feasibility of successful implementation.

Both the US FDA and WHO prequalified RHTs have diagnostic sensitivity and specificity of ≥99% and ≥98%, respectively, and are accurate when testing is performed appropriately. To provide accurate and reliable results to the participants in the PHIA surveys, we sought to ensure adequate training of the survey staff, proper specimen management from standardized specimen collection (either via venous draw or fingerprick), use of suitable collection equipment (e.g., sterile venipuncture kits), and correct labeling and identification. Additionally, the PHIA surveys ensured the availability, proper storage conditions (safe, secure, and temperature-regulated), and timely transportation of RHT to healthcare centers (19). The removal of expired or unused test kits and the monitoring of near-expiry inventory were also critical components of effective stock management. Furthermore, the collection of adequate specimen volume to meet testing requirements, considering potential repeats and following ethical guidelines, led to better results and patient outcomes. For the PHIA surveys, most participants had ~14-mL whole blood specimen collected via venipuncture, stored, and transported in cold chain to a satellite laboratory for additional processing, testing, and storage (12). Adequate and quality specimen volume allowed for repeats, verification, and HIV-positive confirmation of HH testing in the PHIA surveys. The concordance of 5% repeat testing of negative specimens was more than 99.99%. The repeat testing was reduced to 2% in subsequent rounds of the PHIA surveys (12).

In conclusion, the performance of national RHT algorithms in our surveys is optimal. When implemented with high-quality standards, the national HIV testing algorithms will yield accurate and reliable results.
